# Best Alternatives to Cronbach's Alpha Reliability in Realistic Conditions: Congeneric and Asymmetrical Measurements

**DOI:** 10.3389/fpsyg.2016.00769

**Published:** 2016-05-26

**Authors:** Italo Trizano-Hermosilla, Jesús M. Alvarado

**Affiliations:** ^1^Department of Psychology, University of La FronteraTemuco, Chile; ^2^Department of Social Psychology and Methodology, University Autonoma de MadridMadrid, Spain; ^3^Department of Methodology of the Behavioral Sciences, University Complutense de MadridMadrid, Spain

**Keywords:** reliability, alpha, omega, greatest lower bound, asymmetrical measures

## Abstract

The Cronbach's alpha is the most widely used method for estimating internal consistency reliability. This procedure has proved very resistant to the passage of time, even if its limitations are well documented and although there are better options as omega coefficient or the different versions of glb, with obvious advantages especially for applied research in which the ítems differ in quality or have skewed distributions. In this paper, using Monte Carlo simulation, the performance of these reliability coefficients under a one-dimensional model is evaluated in terms of skewness and no tau-equivalence. The results show that omega coefficient is always better choice than alpha and in the presence of skew items is preferable to use omega and glb coefficients even in small samples.

The α coefficient is the most widely used procedure for estimating reliability in applied research. As stated by Sijtsma (2009), its popularity is such that Cronbach (1951) has been cited as a reference more frequently than the article on the discovery of the DNA double helix. Nevertheless, its limitations are well known (Lord and Novick, 1968; Cortina, 1993; Yang and Green, 2011), some of the most important being the assumptions of uncorrelated errors, tau-equivalence and normality.

The assumption of uncorrelated errors (the error score of any pair of items is uncorrelated) is a hypothesis of Classical Test Theory (Lord and Novick, 1968), violation of which may imply the presence of complex multidimensional structures requiring estimation procedures which take this complexity into account (e.g., Tarkkonen and Vehkalahti, 2005; Green and Yang, 2015). It is important to uproot the erroneous belief that the α coefficient is a good indicator of unidimensionality because its value would be higher if the scale were unidimensional. In fact the exact opposite is the case, as was shown by Sijtsma (2009), and its application in such conditions may lead to reliability being heavily overestimated (Raykov, 2001). Consequently, before calculating α it is necessary to check that the data fit unidimensional models.

The assumption of tau-equivalence (i.e., the same true score for all test items, or equal factor loadings of all items in a factorial model) is a requirement for α to be equivalent to the reliability coefficient (Cronbach, 1951). If the assumption of tau-equivalence is violated the true reliability value will be underestimated (Raykov, 1997; Graham, 2006) by an amount which may vary between 0.6 and 11.1% depending on the gravity of the violation (Green and Yang, 2009a). Working with data which comply with this assumption is generally not viable in practice (Teo and Fan, 2013); the congeneric model (i.e., different factor loadings) is the more realistic.

The requirement for multivariant normality is less known and affects both the puntual reliability estimation and the possibility of establishing confidence intervals (Dunn et al., 2014). Sheng and Sheng (2012) observed recently that when the distributions are skewed and/or leptokurtic, a negative bias is produced when the coefficient α is calculated; similar results were presented by Green and Yang (2009b) in an analysis of the effects of non-normal distributions in estimating reliability. Study of skewness problems is more important when we see that in practice researchers habitually work with skewed scales (Micceri, 1989; Norton et al., 2013; Ho and Yu, 2014). For example, Micceri (1989) estimated that about 2/3 of ability and over 4/5 of psychometric measures exhibited at least moderate asymmetry (i.e., skewness around 1). Despite this, the impact of skewness on reliability estimation has been little studied.

Considering the abundant literature on the limitations and biases of the α coefficient (Revelle and Zinbarg, 2009; Sijtsma, 2009, 2012; Cho and Kim, 2015; Sijtsma and van der Ark, 2015), the question arises why researchers continue to use α when alternative coefficients exist which overcome these limitations. It is possible that the excess of procedures for estimating reliability developed in the last century has oscured the debate. This would have been further compounded by the simplicity of calculating this coefficient and its availability in commercial softwares.

The difficulty of estimating the $\rho_{xx^{\prime }}$ reliability coefficient resides in its definition $\rho_{xx^{\prime }}=\sigma_{t}^{2}∕\sigma_{x}^{2}$, which includes the true score in the variance numerator when this is by nature unobservable. The α coefficient tries to approximate this unobservable variance from the covariance between the items or components. Cronbach (1951) showed that in the absence of tau-equivalence, the α coefficient (or Guttman's lambda 3, which is equivalent to α) was a good lower bound approximation. Thus, when the assumptions are violated the problem translates into finding the best possible lower bound; indeed this name is given to the Greatest Lower Bound method (GLB) which is the best possible approximation from a theoretical angle (Jackson and Agunwamba, 1977; Woodhouse and Jackson, 1977; Shapiro and ten Berge, 2000; Sočan, 2000; ten Berge and Sočan, 2004; Sijtsma, 2009). However, Revelle and Zinbarg (2009) consider that ω gives a better lower bound than GLB. There is therefore an unresolved debate as to which of these two methods gives the best lower bound; furthermore the question of non-normality has not been exhaustively investigated, as the present work discusses.

## ω coefficients

McDonald (1999) proposed the ω_*t*_ coefficient for estimating reliability from a factorial analysis framework, which can be expressed formally as:

(1) $$\begin{array}{l} \omega_{t}=\;\frac{{\left(\sum \lambda_{j}\right)}^{2}}{\left[{\left(\sum \lambda_{j}\right)}^{2}+\;\sum \left(1-\lambda_{j}^{2}\right)\right]}=\;\frac{{\left(\sum \lambda_{j}\right)}^{2}}{\left[{\left(\sum \lambda_{j}\right)}^{2}+\left(\sum \psi \right)\right]} \end{array}$$

Where λ_*j*_ is the loading of item *j*, $\lambda_{j}^{2}$ is the communality of item *j* and ψ equates to the uniqueness. The ω_*t*_ coefficient, by including the lambdas in its formulas, is suitable both when tau-equivalence (i.e., equal factor loadings of all test items) exists (ω_*t*_ coincides mathematically with α), and when items with different discriminations are present in the representation of the construct (i.e., different factor loadings of the items: congeneric measurements). Consequently ω_*t*_ corrects the underestimation bias of α when the assumption of tau-equivalence is violated (Dunn et al., 2014) and different studies show that it is one of the best alternatives for estimating reliability (Zinbarg et al., 2005, 2006; Revelle and Zinbarg, 2009), although to date its functioning in conditions of skewness is unknown.

When correlation exists between errors, or there is more than one latent dimension in the data, the contribution of each dimension to the total variance explained is estimated, obtaining the so-called hierarchical ω (ω_*h*_) which enables us to correct the worst overestimation bias of α with multidimensional data (see Tarkkonen and Vehkalahti, 2005; Zinbarg et al., 2005; Revelle and Zinbarg, 2009). Coefficients ω_*h*_ and ω_*t*_ are equivalent in unidimensional data, so we will refer to this coefficient simply as ω.

## Greatest lower bound (GLB)

Sijtsma (2009) shows in a series of studies that one of the most powerful estimators of reliability is GLB—deduced by Woodhouse and Jackson (1977) from the assumptions of Classical Test Theory **(C**_x_ = **C**_t_ + **C**_e_)—an inter-item covariance matrix for observed item scores **C**_x_. It breaks down into two parts: the sum of the inter-item covariance matrix for item true scores **C**_t_; and the inter-item error covariance matrix **C**_e_ (ten Berge and Sočan, 2004). Its expression is:

(2) $$\begin{array}{l} GLB=\;1-\frac{tr\;\left({\text{C}}_{e}\right)}{\sigma_{x}^{2}} \end{array}$$

where $\sigma_{x}^{2}$ is the test variance and tr(***C***_e_) refers to the trace of the inter-item error covariance matrix which it has proved so difficult to estimate. One solution has been to use factorial procedures such as Minimum Rank Factor Analysis (a procedure known as glb.fa). More recently the GLB algebraic (GLBa) procedure has been developed from an algorithm devised by Andreas Moltner (Moltner and Revelle, 2015). According to Revelle (2015a) this procedure adopts the form which is most faithful to the original definition by Jackson and Agunwamba (1977), and it has the added advantage of introducing a vector to weight the items by importance (Al-Homidan, 2008).

Despite its theoretical strengths, GLB has been very little used, although some recent empirical studies have shown that this coefficient produces better results than α (Lila et al., 2014) and α and ω (Wilcox et al., 2014). Nevertheless, in small samples, under the assumption of normality, it tends to overestimate the true reliability value (Shapiro and ten Berge, 2000); however its functioning under non-normal conditions remains unknown, specifically when the distributions of the items are asymmetrical.

Considering the coefficients defined above, and the biases and limitations of each, the object of this work is to evaluate the robustness of these coefficients in the presence of asymmetrical items, considering also the assumption of tau-equivalence and the sample size.

## Methods

### Data generation

The data were generated using R (R Development Core Team, 2013) and RStudio (Racine, 2012) software, following the factorial model:

(3) $$X_{ij}=\sum_{k=1}^{k}\lambda_{jk}F_{k}+\sqrt{\left(1-\sum_{k=1}^{k}\lambda_{jk}^{2}\right)}\times e_{j}$$

where *X*_*ij*_ is the simulated response of subject *i* in item *j*, λ_*jk*_ is the loading of item *j* in Factor *k* (which was generated by the unifactorial model); *F*_*k*_ is the latent factor generated by a standardized normal distribution (mean 0 and variance 1), and *e*_*j*_ is the random measurement error of each item also following a standardized normal distribution.

Skewed items: Standard normal *X*_*ij*_ were transformed to generate non-normal distributions using the procedure proposed by Headrick (2002) applying fifth order polynomial transforms:

(4) $$\begin{array}{l} Y_{ij}=c_{0}+c_{1}X_{ij}+c_{2}X_{ij}^{2}+c_{3}X_{ij}^{3}+c_{4}X_{ij}^{4}+c_{5}X_{ij}^{5} \end{array}$$

The coefficients implemented by Sheng and Sheng (2012) were used to obtain centered, asymmetrical distributions (asymmetry ≈ 1): *c*_0_ = −0.446924, *c*_1_ = 1.242521, *c*_2_ = 0.500764, *c*_3_ = −0.184710, *c*_4_ = −0.017947, *c*_5_ = 0.003159.

### Simulated conditions

To assess the performance of the reliability coefficients (α, ω, GLB and GLBa) we worked with three sample sizes (250, 500, 1000), two test sizes: short (6 items) and long (12 items), two conditions of tau-equivalence (one with tau-equivalence and one without, i.e., congeneric) and the progressive incorporation of asymmetrical items (from all the items being normal to all the items being asymmetrical). In the short test the reliability was set at 0.731, which in the presence of tau-equivalence is achieved with six items with factor loadings = 0.558; while the congeneric model is obtained by setting factor loadings at values of 0.3, 0.4, 0.5, 0.6, 0.7, and 0.8 (see Appendix I). In the long test of 12 items the reliability was set at 0.845 taking the same values as in the short test for both tau-equivalence and the congeneric model (in this case there were two items for each value of lambda). In this way 120 conditions were simulated with 1000 replicas in each case.

### Data analysis

The main analyses were carried out using the *Psych* (Revelle, 2015b) and *GPArotation* (Bernaards and Jennrich, 2015) packets, which allow α and ω to be estimated. Two computerized approaches were used for estimating GLB: glb.fa (Revelle, 2015a) and glb.algebraic (Moltner and Revelle, 2015), the latter worked by authors like Hunt and Bentler (2015).

In order to evaluate the accuracy of the various estimators in recovering reliability, we calculated the Root Mean Square of Error (*RMSE*) and the bias. The first is the mean of the differences between the estimated and the simulated reliability and is formalized as:

(5) $$\begin{array}{l} RMSE=\;\sqrt{\frac{{\sum \left(\hat{\rho }-\;\rho \right)}^{2}}{Nr}} \end{array}$$

where $\hat{\rho }\;$ is the estimated reliability for each coefficient, ρ the simulated reliability and *Nr* the number of replicas. The % bias is understood as the difference between the mean of the estimated reliability and the simulated reliability and is defined as:

(6) $$\begin{array}{l} \%\;bias=\;\frac{\sum \left(\hat{\rho }-\;\rho \right)}{Nr}\;\times \;100 \end{array}$$

In both indices, the greater the value, the greater the inaccuracy of the estimator, but unlike RMSE, the bias may be positive or negative; in this case additional information would be obtained as to whether the coefficient is underestimating or overestimating the simulated reliability parameter. Following the recommendation of Hoogland and Boomsma (1998) values of RMSE < 0.05 and % bias < 5% were considered acceptable.

## Results

The principal results can be seen in Table 1 (6 items) and Table 2 (12 items). These show the RMSE and % bias of the coefficients in tau-equivalence and congeneric conditions, and how the skewness of the test distribution increases with the gradual incorporation of asymmetrical items.

**Table 1:** **RMSE and Bias with tau-equivalence and congeneric condition for 6 items, three sample sizes and the number of skewed items**.

**Skewed items**	**SK**	***n***	**Cond**	**RMSE**				**% bias**			
				**α**	**ω**	**GLB**	**GLBa**	**α**	**ω**	**GLB**	**GLBa**
NORMALITY											
0 items	0.0	250	tau	0.03	0.03	**0.07**	0.04	−0.20	0.00	**6.30**	3.50
			cong	0.03	0.02	**0.07**	0.04	−1.60	−0.10	**6.10**	3.40
		500	tau	0.02	0.02	**0.06**	0.03	0.00	0.00	**5.70**	2.50
			cong	0.02	0.02	**0.06**	0.03	−1.50	0.00	**5.50**	2.50
		1000	tau	0.01	0.01	**0.06**	0.02	−0.10	−0.10	4.90	1.70
			cong	0.02	0.01	**0.06**	0.02	−1.50	−0.10	**5.00**	1.70
2 items	0.3	250	tau	**0.06**	**0.06**	0.04	0.03	−**5.50**	−**5.00**	2.70	−0.80
			cong	**0.06**	0.04	**0.05**	0.03	−**5.40**	−3.00	3.90	0.90
		500	tau	**0.06**	**0.05**	0.04	0.03	−**5.20**	−4.90	1.80	−1.90
			cong	**0.06**	0.03	0.04	0.02	−**5.10**	−2.80	3.20	−0.10
		1000	tau	**0.06**	**0.05**	0.03	0.03	−**5.20**	−**5.00**	1.00	−2.70
			cong	**0.05**	0.03	0.04	0.02	−**5.20**	−2.90	2.40	−1.00
4 items	0.6	250	tau	**0.11**	**0.10**	0.04	**0.06**	−**10.00**	−**9.60**	−0.80	−4.70
			cong	**0.11**	**0.09**	0.04	**0.05**	−**10.70**	−**8.10**	0.20	−3.50
		500	tau	**0.10**	**0.10**	0.04	**0.06**	−**9.70**	−**9.40**	−1.60	−**5.80**
			cong	**0.11**	**0.08**	0.03	**0.05**	−**10.50**	−**8.00**	−0.30	−4.60
		1000	tau	**0.10**	**0.10**	0.04	**0.07**	−**9.60**	−**9.50**	−2.50	−**6.80**
			cong	**0.08**	**0.05**	0.03	0.04	−**7.70**	−**5.20**	0.80	−3.10
All items	0.9	250	tau	**0.14**	**0.14**	**0.06**	**0.09**	−**13.10**	-**12.80**	−3.00	−**7.50**
			cong	**0.15**	**0.13**	**0.05**	**0.08**	−**13.80**	−**11.80**	−2.50	−**6.80**
		500	tau	**0.13**	**0.13**	**0.06**	**0.09**	−**12.70**	−**12.50**	−4.10	−**8.80**
			cong	**0.14**	**0.12**	**0.05**	**0.09**	−**13.50**	−**11.70**	−3.40	−**8.00**
		1000	tau	**0.13**	**0.13**	**0.06**	**0.10**	−**12.70**	-**12.60**	−**5.00**	−**9.90**
			cong	**0.14**	**0.12**	**0.06**	**0.09**	−**13.40**	−**11.60**	−4.10	−**9.00**

RMSE, Root Mean Square of Error; SK, test skewness; n, sample size; cond, Condition; tau, tau-equivalent model; cong, Congeneric model; α, coefficient alpha; ω, coefficient omega; GLB, Greatest Lower Bound (GLB.fa); GLBa, Greatest Lower Bound (GLB.algebraic); bold RMSE ≥ 0.05 or % bias ≥ |5%|

**Table 2:** **RMSE and Bias with tau-equivalence and congeneric condition for 12 items, three sample sizes and the number of skewed items**.

**Skewed items**	**SK**	***n***	**Cond**	**RMSE**				**% bias**			
				**α**	**ω**	**GLB**	**GLBa**	**α**	**ω**	**GLB**	**GLBa**
NORMALITY											
0 items	0.0	250	tau	0.02	0.02	**0.05**	0.04	−0.10	−0.10	4.80	3.90
			cong	0.02	0.01	**0.05**	0.04	−0.90	−0.10	4.40	3.80
		500	tau	0.01	0.01	0.04	0.03	−0.10	−0.10	4.20	2.80
			cong	0.01	0.01	0.04	0.03	−0.80	−0.10	3.70	2.70
		1000	tau	0.01	0.01	0.04	0.02	0.00	0.00	3.70	2.00
			cong	0.01	0.01	0.03	0.02	−0.80	0.00	3.10	2.00
2 items	0.2	250	tau	0.03	0.02	0.04	0.03	−1.90	−1.70	3.60	2.60
			cong	0.03	0.02	0.04	0.03	−2.00	−1.00	3.70	3.10
		500	tau	0.02	0.02	0.03	0.02	−1.80	−1.70	2.90	1.40
			cong	0.02	0.01	0.03	0.02	−2.00	−1.00	3.00	1.90
		1000	tau	0.02	0.02	0.03	0.01	−1.80	−1.70	2.40	0.60
			cong	0.02	0.01	0.03	0.01	−1.90	−1.00	2.30	1.10
4 items	0.3	250	tau	0.04	0.04	0.03	0.02	−3.60	−3.50	2.40	1.40
			cong	0.04	0.03	0.03	0.02	−3.90	−2.80	2.50	1.80
		500	tau	0.04	0.04	0.02	0.01	−3.50	−3.40	1.70	0.10
			cong	0.04	0.03	0.02	0.01	−3.80	−2.70	1.70	0.60
		1000	tau	0.04	0.04	0.02	0.01	−3.50	−3.40	1.00	−0.80
			cong	0.04	0.03	0.02	0.01	−3.80	−2.70	1.00	−0.30
6 items	0.5	250	tau	**0.06**	**0.06**	0.02	0.02	−**5.40**	−**5.20**	1.30	0.20
			cong	**0.06**	**0.06**	0.02	0.02	−**6.10**	−**5.20**	1.00	0.30
		500	tau	**0.05**	**0.05**	0.02	0.02	−**5.20**	−**5.10**	0.40	−1.20
			cong	**0.06**	**0.05**	0.02	0.02	−**6.00**	−**5.10**	−0.10	−1.10
		1000	tau	**0.05**	**0.05**	0.02	0.02	−**5.20**	−**5.10**	−0.40	−2.20
			cong	**0.06**	**0.05**	0.02	0.02	−**5.90**	−**5.10**	−0.80	−2.10
8 items	0.6	250	tau	**0.07**	**0.07**	0.02	0.02	−**6.90**	−**6.70**	0.20	−1.00
			cong	**0.08**	**0.07**	0.02	0.02	−**7.30**	−**6.20**	0.20	−0.50
		500	tau	**0.07**	**0.07**	0.02	0.03	−**6.80**	−**6.70**	−0.70	−2.50
			cong	**0.07**	**0.06**	0.02	0.02	−**7.20**	−**6.10**	−0.80	−1.90
		1000	tau	**0.07**	**0.07**	0.02	0.04	−**6.70**	−**6.60**	−1.50	−3.60
			cong	**0.07**	**0.06**	0.02	0.03	−**7.10**	−**6.10**	−1.70	−3.00
10 items	0.8	250	tau	**0.09**	**0.09**	0.03	0.03	−**8.30**	−**8.10**	−0.70	−2.00
			cong	**0.09**	**0.08**	0.02	0.03	−**8.80**	−**7.70**	−0.90	−1.60
		500	tau	**0.08**	**0.08**	0.03	0.04	−**8.10**	−**8.00**	−1.70	−3.60
			cong	**0.09**	**0.08**	0.03	0.04	−**8.60**	−**7.60**	−1.60	−3.10
		1000	tau	**0.08**	**0.08**	0.03	**0.05**	−**8.10**	−**8.00**	−2.40	−4.80
			cong	**0.09**	**0.08**	0.03	0.04	−**8.60**	−**7.60**	−2.40	−4.30
All items	1.0	250	tau	**0.10**	**0.10**	0.03	0.04	−**9.40**	−**9.20**	−1.40	−2.80
			cong	**0.10**	**0.09**	0.03	0.03	−**9.50**	−**8.50**	−1.30	−2.30
		500	tau	**0.09**	**0.09**	0.03	**0.05**	−**9.20**	−**9.10**	−2.30	−4.60
			cong	**0.10**	**0.09**	0.03	0.04	−**9.40**	−**8.40**	−2.30	−3.90
		1000	tau	**0.09**	**0.09**	0.04	**0.06**	−**9.20**	−**9.10**	−3.20	−**5.90**
			cong	**0.09**	**0.08**	0.04	**0.05**	−**9.30**	−**8.30**	−3.20	−**5.20**

*RMSE, Root Mean Square of Error; SK, test skewness; n, sample size; cond, Condition; tau, tau-equivalent model; cong, Congeneric model; α, coefficient alpha; ω, coefficient omega; GLB, Greatest Lower Bound (GLB.fa); GLBa, Greatest Lower Bound (GLB.algebraic); bold RMSE ≥ 0.05 or % bias ≥ |5%|*.

Only under conditions of tau-equivalence and normality (skewness < 0.2) is it observed that the α coefficient estimates the simulated reliability correctly, like ω. In the congeneric condition ω corrects the underestimation of α. Both GLB and GLBa present a positive bias under normality, however GLBa shows approximatively ½ less % bias than GLB (see Table 1). If we consider sample size, we observe that as the test size increases, the positive bias of GLB and GLBa diminishes, but never disappears.

In asymmetrical conditions, we see in Table 1 that both α and ω present an unacceptable performance with increasing RMSE and underestimations which may reach bias > 13% for the α coefficient (between 1 and 2% lower for ω). The GLB and GLBa coefficients present a lower RMSE when the test skewness or the number of asymmetrical items increases (see Tables 1, 2). The GLB coefficient presents better estimates when the test skewness value of the test is around 0.30; GLBa is very similar, presenting better estimates than ω with an test skewness value around 0.20 or 0.30. However, when the skewness value increases to 0.50 or 0.60, GLB presents better performance than GLBa. The test size (6 or 12 ítems) has a much more important effect than the sample size on the accuracy of estimates.

## Discussion

In this study four factors were manipulated: tau-equivalence or congeneric model, sample size (250, 500, and 1000), the number of test items (6 and 12) and the number of asymmetrical items (from 0 asymmetrical items to all the items being asymmetrical) in order to evaluate robustness to the presence of asymmetrical data in the four reliability coefficients analyzed. These results are discussed below.

In conditions of tau-equivalence, the α and ω coefficients converge, however in the absence of tau-equivalence (congeneric), ω always presents better estimates and smaller RMSE and % bias than α. In this more realistic condition therefore (Green and Yang, 2009a; Yang and Green, 2011), α becomes a negatively biased reliability estimator (Graham, 2006; Sijtsma, 2009; Cho and Kim, 2015) and ω is always preferable to α (Dunn et al., 2014). In the case of non-violation of the assumption of normality, ω is the best estimator of all the coefficients evaluated (Revelle and Zinbarg, 2009).

Turning to sample size, we observe that this factor has a small effect under normality or a slight departure from normality: the RMSE and the bias diminish as the sample size increases. Nevertheless, it may be said that for these two coefficients, with sample size of 250 and normality we obtain relatively accurate estimates (Tang and Cui, 2012; Javali et al., 2011). For the GLB and GLBa coefficients, as the sample size increases the RMSE and the bias tend to diminish; however they maintain a positive bias for the condition of normality even with large sample sizes of 1000 (Shapiro and ten Berge, 2000; ten Berge and Sočan, 2004; Sijtsma, 2009).

For the test size we generally observe a higher RMSE and bias with 6 items than with 12, suggesting that the higher the number of items, the lower the RMSE and the bias of the estimators (Cortina, 1993). In general the trend is maintained for both 6 and 12 items.

When we look at the effect of progressively incorporating asymmetrical items into the data set, we observe that the α coefficient is highly sensitive to asymmetrical items; these results are similar to those found by Sheng and Sheng (2012) and Green and Yang (2009b). Coefficient ω presents similar RMSE and bias values to those of α, but slightly better, even with tau-equivalence. GLB and GLBa are found to present better estimates when the test skewness departs from values close to 0.

Considering that in practice it is common to find asymmetrical data (Micceri, 1989; Norton et al., 2013; Ho and Yu, 2014), Sijtsma's suggestion (2009) of using GLB as a reliability estimator appears well-founded. Other authors, such as Revelle and Zinbarg (2009) and Green and Yang (2009a), recommend the use of ω, however this coefficient only produced good results in the condition of normality, or with low proportion of skewness items. In any case, these coefficients presented greater theoretical and empirical advantages than α. Nevertheless, we recommend researchers to study not only punctual estimates but also to make use of interval estimation (Dunn et al., 2014).

These results are limited to the simulated conditions and it is assumed that there is no correlation between errors. This would make it necessary to carry out further research to evaluate the functioning of the various reliability coefficients with more complex multidimensional structures (Reise, 2012; Green and Yang, 2015) and in the presence of ordinal and/or categorical data in which non-compliance with the assumption of normality is the norm.

## Conclusion

When the total test scores are normally distributed (i.e., all items are normally distributed) ω should be the first choice, followed by α, since they avoid the overestimation problems presented by GLB. However, when there is a low or moderate test skewness GLBa should be used. GLB is recommended when the proportion of asymmetrical items is high, since under these conditions the use of both α and ω as reliability estimators is not advisable, whatever the sample size.

## Author contributions

Development of the idea of research and theoretical framework (IT, JA). Construction of the methodological framework (IT, JA). Development of the R language syntax (IT, JA). Data analysis and interpretation of data (IT, JA). Discussion of the results in light of current theoretical background (JA, IT). Preparation and writing of the article (JA, IT). In general, both authors have contributed equally to the development of this work.

## Funding

The first author disclosed receipt of the following financial support for the research, authorship, and/or publication of this article: IT received financial support from the Chilean National Commission for Scientific and Technological Research (CONICYT) “Becas Chile” Doctoral Fellowship program (Grant no: 72140548).

### Conflict of interest statement

The authors declare that the research was conducted in the absence of any commercial or financial relationships that could be construed as a potential conflict of interest.

## Appendix I

R syntax to estimate reliability coefficients from Pearson's correlation matrices. The correlation values outside the diagonal are calculated by multiplying the factor loading of the items: (1) tau-equivalent model they are all equal to 0.3114 (λ_*i*_λ_*j*_ = 0.558 × 0.558 = 0.3114) and (2) congeneric model they vary as a function of the different factor loading (e.g., the matrix element a_1, 2_ = λ_1_λ_2_ = 0.3 × 0.4 = 0.12). In both examples the true reliability is 0.731.

**# Example 1. Tau-equivalent model with λ = 0.558 for the six items**

> library(psych)

> library(Rcsdp)

> Cr <-matrix(c(1.00, 0.3114, 0.3114, 0.3114, 0.3114, 0.3114,

                      0.3114, 1.00, 0.3114, 0.3114, 0.3114, 0.3114,

                      0.3114, 0.3114, 1.00, 0.3114, 0.3114, 0.3114,

                      0.3114, 0.3114, 0.3114, 1.00, 0.3114, 0.3114,

                      0.3114, 0.3114, 0.3114, 0.3114, 1.00, 0.3114,

                      0.3114, 0.3114, 0.3114, 0.3114, 0.3114, 1.00),

                      ncol = 6)

> omega(Cr,1)$alpha # standardized Cronbach's α

   [1] 0.731

> omega(Cr,1)$omega.tot # coefficient ω total

   [1] 0.731

> glb.fa(Cr)$glb # GLB factorial procedure

   [1] 0.731

> glb.algebraic(Cr)$glb # GLB algebraic procedure

   [1] 0.731

**# Example 2. Congeneric model with λ**_1_ **= 0.3**, **λ**_2_ **= 0.4**, **λ**_3_ **= 0.5**, **λ**_4_ **= 0.6**, **λ**_5_ **= 0.7**, **λ**_6_ **= 0.8**

> Cr <-matrix(c(1.00, 0.12, 0.15, 0.18, 0.21, 0.24,

                      0.12, 1.00, 0.20, 0.24, 0.28, 0.32,

                      0.15, 0.20, 1.00, 0.30, 0.35, 0.40,

                      0.18, 0.24, 0.30, 1.00, 0.42, 0.48,

                      0.21, 0.28, 0.35, 0.42, 1.00, 0.56,

                      0.24, 0.32, 0.40, 0.48, 0.56, 1.00),

                      ncol = 6)

> omega(Cr,1)$alpha # standardized Cronbach's α

   [1] 0.717

> omega(Cr,1)$omega.tot # coefficient ω total

   [1] 0.731

> glb.fa(Cr)$glb # GLB factorial procedure

   [1] 0.754

> glb.algebraic(Cr)$glb # GLB algebraic procedure

   [1] 0.731
